# Protocol for Facile Synthesis of Fmoc-N-Me-AA-OH Using 2-CTC Resin as Temporary and Reusable Protecting Group

**DOI:** 10.3390/mps6060110

**Published:** 2023-11-13

**Authors:** Tanya Román, Gerardo Acosta, Constanza Cárdenas, Beatriz G. de la Torre, Fanny Guzmán, Fernando Albericio

**Affiliations:** 1 Núcleo Biotecnología Curauma, Pontificia Universidad Católica de Valparaíso, Valparaíso 2373223, Chile; tanya.roman.21@gmail.com (T.R.); constanza.cardenas@pucv.cl (C.C.); 2 Doctorado en Biotecnología, Pontificia Universidad Católica de Valparaíso, Universidad Técnica Federico Santa María, Valparaíso 2373223, Chile; 3 Department of Organic Chemistry and CIBER-BBN, Networking Centre on Bioengineering, Biomaterials and Nanomedicine, University of Barcelona, 08028 Barcelona, Spain; gerardoacosta@ub.edu; 4 Institute for Advanced Chemistry of Catalonia (IQAC-CSIC), Jordi Girona 18-26, 08034 Barcelona, Spain; 5 KwaZulu-Natal Research Innovation and Sequencing Platform (KRISP), School of Laboratory Medicine and Medical Sciences, College of Health Sciences, University of KwaZulu-Natal, Durban 4041, South Africa; garciadelatorreb@ukzn.ac.za; 6 School of Chemistry and Physics, University of KwaZulu-Natal, Durban 4001, South Africa

**Keywords:** 2-CTC resin, N-methylation, alpha and beta amino acids

## Abstract

One approach to enhance the bioavailability and half-life of peptides in vivo is through N-methylation of one or more of the amino acids within the peptide sequence. However, commercially available Fmoc-N-Me-AA-OHs are limited and often expensive. In this study, a solid-phase synthesis method for Fmoc-N-Me-AA-OH was developed using a 2-chlorotrityl chloride (2-CTC) resin as a temporary protective group for the carboxylic acid strategy. Two strategies for the alkylation step were compared, employing either dimethyl sulfate or methyl iodide in the Biron−Kessler method. In this work we tested the protocol with two amino acids: Fmoc-Thr(tBu)-OH and Fmoc-βAla-OH. The first one is an alpha amino acid, very hindered and with the amine group directly influenced by the electronic effects of the carboxy group, whereas in Fmoc-βAla-OH, the presence of a methylene group weakens this influence due to the intervening carbon atoms. The desired amino acids, Fmoc-N-Me-Thr(tBu)-OH and Fmoc-N-Me-βAla-OH, were synthesized by both strategies with high yield and purity.

## 1. Introduction

Synthetic peptides are compounds with broad applications in research and drug development [1,2,3]. There are currently many peptide-type compounds on the market [4,5], and they are extensively studied in various contexts, including their potential use as alternatives for overcoming antibiotic resistance [6,7]. However, one limitation of peptides as drug-like compounds is their relatively short half-life, which affects their bioavailability. To address this, N-methylation of at least one amino acid within the peptide sequence has been commonly employed. This modification enhances peptide stability and bioavailability, and can impart other beneficial properties, such as improved cell permeability and enhanced interaction with hydrophobic targets [8,9,10,11,12]; arginine methylation has also been reported as a modulator in the self-assembly process of peptides, with applications in biomedicine [13]. A strategy to achieve N-methylation involves obtaining a protected methylated amino acid, known as Fmoc-N-Me-AA-OH, which is subsequently incorporated into the synthetic peptide sequence.

**Scheme 1:**
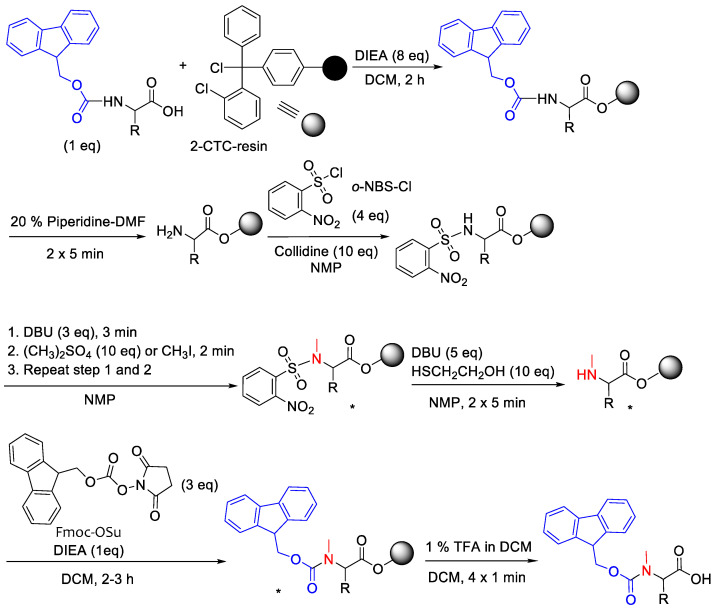
Solid-phase synthesis of Fmoc-N-Me-AA-OH using 2-CTC resin as temporary protecting group. * The progress of the reaction was monitored in each step by a mini-cleavage (Section 2.3.3).

One of the most effective methods for N-methylation of amino acids is the Biron−Kessler method in solid-phase [14,15,16,17], which is based on the earlier work of Fukuyama in solution [18] and Miller and Scanlan in solid-phase [19]. The Biron−Kessler method involves the protection of the α-amino group with 2-nitrobenzenesulfonyl (*o*-NBS), which renders the remaining NH group acidic and susceptible to methylation. Removal of the *o*-NBS group releases the free NMe amine, which can then be protected again using a more suitable protective group, or acylated with a new protected amino acid (refer to Scheme 1 for the strategy). Various reagents are available for the crucial methylation step, with dimethyl sulfate and methyl iodide being commonly used in peptide synthesis [20].

In this study, the solid-phase synthesis of Fmoc-N-Me-AA-OH was developed, comparing methylation with dimethyl sulfate or with methyl iodide. A similar strategy has been published by Yang and Chiu [21] and Di Gioia et al. [22]. The 2-chlorotrityl chloride resin (2-CTC resin) was employed as a temporary protecting group for the carboxylic acid strategy. Methylation experiments were conducted using two test amino acids: Fmoc-Thr(tBu)-OH and Fmoc-βAla-OH. The first one is a very hindered alpha amino acid, with the amine group directly influenced by the electronic effects of the carboxy group, whereas in Fmoc-βAla-OH the presence of a methylene group weakens this influence due to the intervening carbon atoms.

On the other hand, the present study emphasizes the utilization of the 2-CTC resin, which provides the advantage of reusability across multiple cycles of peptide synthesis [23]. The temporary protecting group provided by the resin enables efficient methylation of the amino acid without interfering with the subsequent steps of synthesis. Reusing the resin enhances the cost-effectiveness of the synthesis process. This approach has the potential for scalability to produce larger quantities of Fmoc-N-Me-AA-OH, thanks to the high level of substitutions achievable with the 2-CTC resin. An additional advantage is that the methylation reaction is brought to completion without any racemization occurring. This contributes to the advancement of research and development in the field of drug discovery.

## 2. Experimental Design

Dimethyl sulfate or methyl iodide were employed as methylating reagents. The 2-CTC resin was used to temporarily protect the carboxylic group during peptide synthesis, with Fmoc-Thr(tBu)-OH and Fmoc-βAla-OH, being incorporated onto the resin for this purpose. The Fmoc group was removed and the liberated α-amino group was protected again using o-nitrobenzenesulfonyl chloride (*o*-NBS-Cl) to form the *o*-NBS protecting group. The sulfonyl group and the nitro group in the ortho position of *o*-NBS contributed to acidifying the NH of the amino acid. Direct N alkylation was then carried out by treating the resin-bound amino acid with 1,8-diazabicyclo [5.4.0]undec-7-ene (DBU) along with either dimethyl sulfate or methyl iodide as the alkylating agent.

After the methylation step, the *o*-NBS protecting group was removed using mercaptoethanol, and the free N-terminal group was protected again with Fmoc-OSu to form the Fmoc-protected amino acid. Finally, the amino acid was cleaved from the resin using 1% trifluoroacetic acid (TFA), ensuring that the side chain remained protected (Scheme 1). The resulting methylated amino acid was ready to be incorporated into the synthesis process of the target peptide.

### 2.1. Materials and Reagents

#### 2.1.1. Resin

- 2-Chlorotrityl chloride resin (2-CTC resin) (loading 1.59 mmol/g)

#### 2.1.2. Fmoc-Amino Acids-OH

- Fmoc-L-βAla-OH
- Fmoc-L-Thr(tBu)-OH

#### 2.1.3. Solvents Synthesis Grade

- N-Methyl-2-pyrrolidine (NMP)
- Methanol (MeOH)
- Anhydrous dichloromethane (anh. DCM)

#### 2.1.4. Reagents to Activate the Resin

- Thionyl chloride
- N-Ethyl diisopropylamine (DIEA)
- Sodium hydroxide (NaOH)

#### 2.1.5. N-Methylation Reagent

- 2-Nitrobenzenesulfonyl chloride (o-NBS-Cl)
- 1,8-Diazabicyclo [5.4.0]undec-7-ene (DBU)
- Dimethyl sulfate
- Methyl iodide
- 2,4,6-Trimethylpyridine (Collidine)
- 2-Mercaptoethanol
- 9-Fluorenylmethyl-succinimidyl carbonate (Fmoc-OSu)
- Piperidine

#### 2.1.6. Cleavage Reagent

- Trifluoroacetic acid (TFA)
- Dichloromethane (DCM)

A syringe of propylene of 2 mL for 150 mg of resin with a propylene filter was used as reactor; it was attached to a vacuum pump for its drainage.

The resin and the amino acids were purchased from Iris Biotech GmbH (Marktred-witz, Germany). The solvent and the reagent to activate the resin were purchased from Merck KGaA (Darmstadt, Germany).

### 2.2. Preparation of Solutions

#### 2.2.1. *o*-NBS-Cl (Solution A)

Four equivalents (Eq) of *o*-NBS-Cl and ten Eq of collidine in NMP: to prepare 30 mL of Solution A we weighed 1.77 g *o*-NBS-Cl, added 10 mL of NMP, and dissolved. Then, we added 2.64 mL of collidine and filled the container up to 30 mL with NMP.

#### 2.2.2. N-Methylation Solution

Manipulation of methylating agents was always carried out under a hood and the researcher wore gloves, goggles, and mask.

##### DBU Solution (Solution B)

Three Eq of DBU in NMP: to prepare 30 mL of Solution B we poured 897 μL of DBU and filled the container up to 30 mL with NMP.

##### Methylating Agent

Dimethyl sulfate or methyl iodide were used in this step.

- Dimethyl sulfate solution (Solution C1)

Ten Eq of dimethyl sulfate in NMP: to prepare 30 mL of Solution C1 we poured 1.89 mL of dimethyl sulfate and filled the container up to 30 mL with NMP.

- Methyl iodide solution (Solution C2)

Ten Eq of methyl iodide in NMP: to prepare 30 mL of Solution C2 we poured 978 μL of methyl iodide in and filled the container up to 30 mL with NMP.

##### 2.2.3. *o*-NBS-Cl Deprotection Solution (Solution D)

Ten Eq of 2-mercaptoethanol and five Eq DBU in NMP: to prepare 30 mL of Solution D we poured 1.4 mL of 2-mercaptoethanol, added 15 mL of NMP, and mixed. Then, we added 1.5 mL of DBU and filled the container up to 30 mL with NMP.

#### 2.2.4. Fmoc Protection Solution (Solution E)

Three Eq of Fmoc-OSu and one Eq of DIEA in DCM: to prepare 2 mL of Solution E we weighed 1.0 g Fmoc-OSu, added 2 mL of DCM, dissolved it, incorporated it into the reactor, and immediately added 174.3 μL of DIEA.

#### 2.2.5. Cleavage Solution

For 1% TFA in DCM. For 50 mL of cleavage solution, we poured 500 µL of TFA and fille the container up to 50 mL with DCM.

### 2.3. Analytical Tests

**Figure 1:**
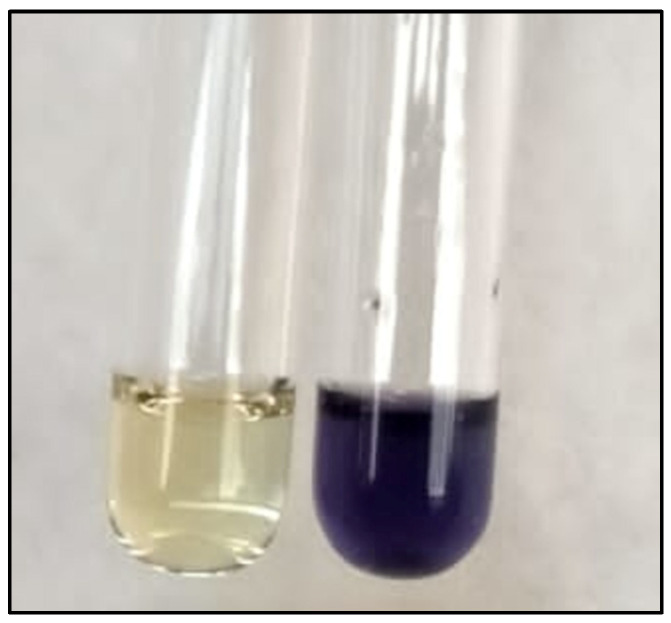
**Ninhydrin test.** Left side: negative result (absence of free amines). Right side: positive result (presence of free amines).

**Figure 2:**
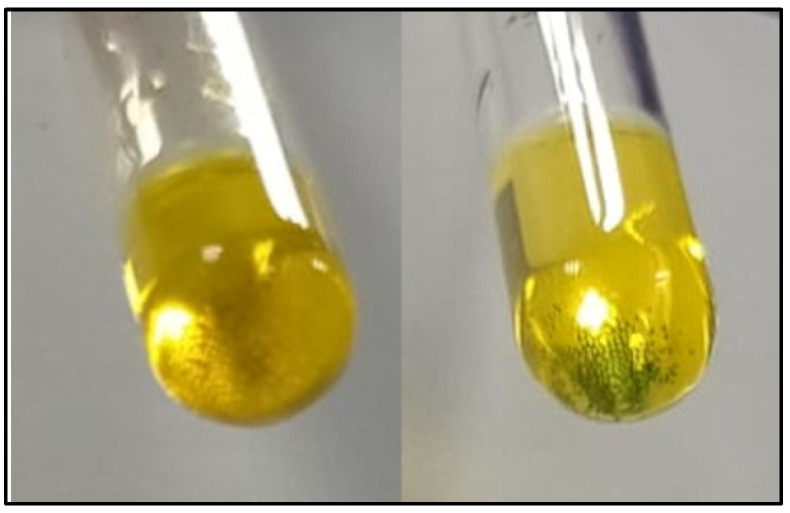
**Chloranil test.** Left side: negative result, revealing the yellow colored resin beads (absence of free secondary amines). Right side: positive result, revealing the blue-green colored resin beads (presence of free secondary amines).

The progress of the reactions was monitored using two colorimetric methods: the ninhydrin test (Figure 1) and the chloranil test (Figure 2). These tests allowed us to determine the presence or absence of free amines. Additionally, an aliquot of the resin (approximately 5 mg) was cleaved with 1% trifluoroacetic acid (TFA) and analysed using liquid chromatography-mass spectrometry (LC-MS). This analysis provided further insight into the reaction progress and allowed for the identification and characterization of the synthesized compounds.

#### 2.3.1. Ninhydrin Test [24,25]

We transferred a small amount of dry resin into a small vial via disposable pipette. We added 3 drops of Solution I (Sol I: 1 part of phenol-ethanol and 10 parts of KCN- pyridine) and 1 drop of Solution II (Sol II: 2.5 g of ninhydrin in 50 mL of absolute ethanol). We heated this at 100 °C for 5 min. Free primary amines indication was noted when the resin beads turned blue and the solution turned blue as well.

#### 2.3.2. Chloranil Test [26]

We transferred a small amount of dry resin into a small vial using a spatula. We added 5 drops of a saturated solution of chloranil to toluene, added 10 drops of acetone, and let this remain at room temperature for 5–10 min. Free secondary amines indication was noted when the resin beads turned blue-green. Please be aware that the chloranil test is less sensitive than the ninhydrin test.

#### 2.3.3. Mini-Cleavage

We transferred a small amount of dry resin (5 mg) to an Eppendorf tube. Then, we added 500 μL of cleavage solution, evaporated the solution after 30 min, added 200 μL of H_2_O/ACN (1:1), and mixed. Next, we centrifuged for 2 min at 2000 rpm and took an aliquot (20 μL) for analysis by LC-MS.

### 2.4. Synthetic Process

#### 2.4.1. 2-CTC Resin Activation

**Figure 3:**
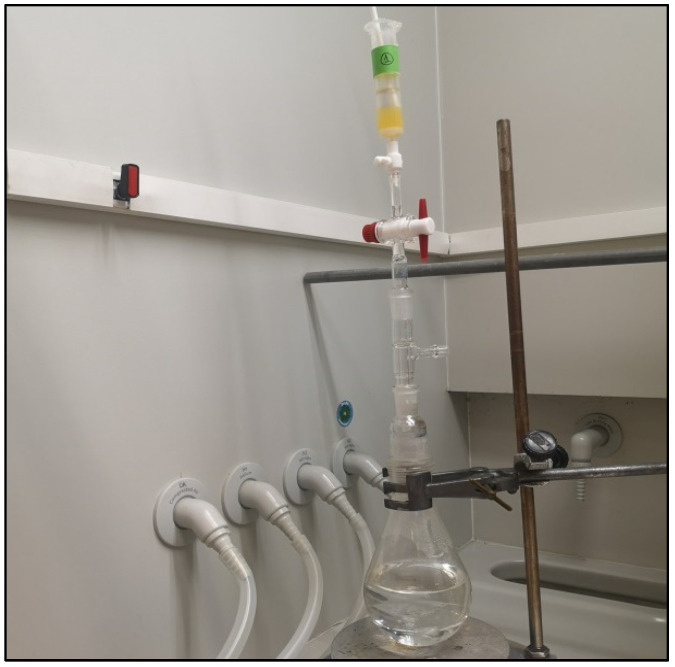
The 2-CTC resin activation solution was discarded on a balloon containing a basic solution to neutralize (NaOH 1 M).

The pre-weighed 2-CTC resin (150 mg, as shown in Table 1) was added to the reactor and allowed to swell in anh. DCM for 10 min. The solvent was then discarded. For every 1 g of 2-CTC resin, 10 mL of a mixture of thionyl chloride and anh. DCM (1:1) was added to the reactor. The mixture was shaken for 1 h. Subsequently, the solution was discarded into a trap containing 1 M NaOH solution (Figure 3) to neutralize any excess reagent. The resin was then thoroughly washed five times with DCM (10 mL/g resin) to remove any remaining impurities or reagents.

#### 2.4.2. Fmoc-Amino Acid C-Carboxylic Acid Protection and Fmoc-AA-O-CTC Resin

**Figure 4:**
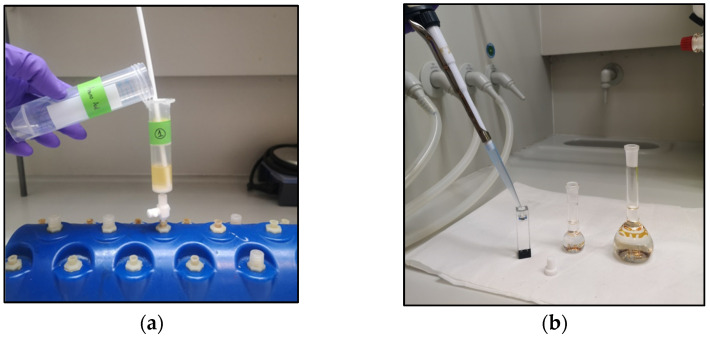
(**a**) Incorporation of Fmoc-amino acid into the 2-CTC resin; (**b**) preparation of the solution in a quartz cuvette for measurement of absorbance at 290 nm in a spectrophotometer.

Anh. DCM was used as solvent and DIEA was used as base in the synthesis process. Three equivalents (Eq) of Fmoc-amino acid were weighed and dissolved in the smallest possible volume of anh. DCM (1.5 mL/g resin). The resulting solution was then incorporated into the reactor (Figure 4a). Next, nine equivalents of DIEA were immediately added to the mixture, and the reaction mixture was stirred for 2 h.

To cap the free amino groups, without draining the solution beforehand, methanol (MeOH) was added (0.8 mL/g resin) and the mixture was shaken for 30 min. Subsequently, the solution was discarded, and the resin-bound amino acid was washed three times with DCM, followed by two washings with DMF and two additional washings with DCM.

#### 2.4.3. H-AA-O-CTC Resin and Loading Quantification

After incorporating the Fmoc-amino acid onto the 2-CTC resin, it was important to determine the degree of substitution (load) of the resin to calculate the appropriate amounts of reagents to be used in subsequent steps.

To remove the Fmoc protecting group of the first incorporated amino acid, 1 mL of 20% piperidine in DMF was added and allowed to react for 5 min. The drained solvent was collected in a 50 mL volumetric flask. This deprotection step was repeated for an additional 5 min, and the drained solvent was also collected in the same 50 mL volumetric flask. The resin was then washed twice with 1 mL of DMF, and the drained solvents from the washings were combined with the previously collected solvent in the 50 mL volumetric flask. The flask was then filled up to 50 mL with DMF. An aliquot of this solution, ranging from 50 to 500 µL depending on the amount of deprotected resin, was taken and transferred to a 25 mL flask. For example, for 100 mg of total resin, 500 µL of the solution was taken, and for 1 g of total resin, 50 µL of the solution was taken. The 25 mL flask was then filled up with DMF. The absorbance of this solution was measured in a spectrophotometer at 290 nm using quartz cuvettes; DMF was used as blank (Figure 4b). The resin load was calculated using Equation (1) [27]:

(1) Loading=(Abs×DF)/(ε×1×g)

where, Abs = measured absorbance at 290 nm; DF = dilution factor*; ε = extinction coefficient; 1 = light path length; g = grams of resin used. The extinction coefficient of dibenzofulvene at 290 nm was used (5800 mol^−1^·cm^−1^).

The dilution factor, in this case, corresponds to the dilution at which the piperidine solution used was subjected, according to the aliquot taken. For example, if piperidine was added to a 50 mL volume flask and then a 100 µL aliquot was taken and added to a 25 mL volume flask, the dilution factor would be 50 × 25/0.1 = 12,500.

#### 2.4.4. Resin Swelling

The resin was swollen with NMP at a ratio of 5 mL/g resin for 15 min, and then the solvent was carefully discarded.

#### 2.4.5. *o*-NBS-Cl Protection Activation

**Figure 5:**
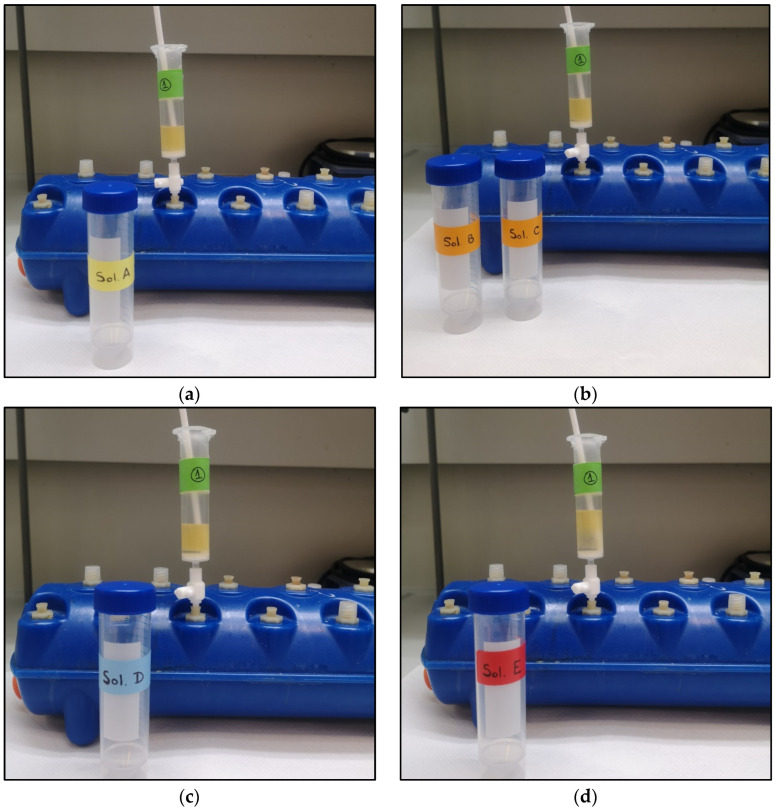
(**a**) Incorporation of Solution A into the 2-CTC resin; (**b**) incorporation of Solution B and after 3 min incorporation of the Solution C into the 2-CTC resin; (**c**) incorporation of Solution D into the 2-CTC resin; (**d**) incorporation of Solution E into the 2-CTC resin.

**Figure 6:**
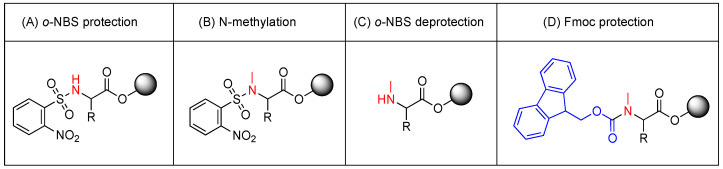
The structure of each molecule obtained after at each methylation step.

The initial step involved adding Solution A in an amount of 10 mL per gram of resin to fully cover the resin, and the mixture was stirred for 15 min (Figure 5a). Subsequently, Solution A was drained from the reaction vessel and the resin was washed five times with NMP (5 mL/g resin). The ninhydrin test (as described in Note 1) was performed to confirm the complete protection of the resin (Figure 6A).

Note 1: The evaluation of the presence of free amine to verify a complete reaction with *o*-NBS-Cl was determined with the ninhydrin test [24,25]. The results comprise a yellow solution: complete protection (Continue with Section 2.4.6); or a blue solution: incomplete protection (repeat steps (Section 2.4.5)).

#### 2.4.6. N-Methylation

We added 10 mL/g of Solution B resin and shook this for 3 min. Next, we added 10 mL/g of Solution C resin (either Solution C1 prepared with dimethyl sulfate or Solution C2 prepared with methyl iodide) and shook this for 2 more minutes (Figure 5b). Subsequently, the mixture of Solution B and C was drained and washed twice with NMP. The same procedure was repeated twice (Solution B, Solution C, and wash). Finally, a mini-cleavage was performed prior to analysis by LC-MS to assess the complete amino acid methylation (Note 2) (Figure 6B).

In this case, solution C corresponds to Solution C1 prepared with dimethyl sulfate or Solution C2 prepared with methyl iodide. Both solutions were prepared and used independently for comparison in the N-methylation step.

Note 2: The mini-cleavage or cleavage of a minimal resin-amino acid amount, is recommended to assess complete amino acid methylation. If non-methylated amino acid is still present, repeat steps (Section 2.4.6) again. In the case of observing complete methylation, continue with Section 2.4.7.

#### 2.4.7. *o*-NBS Removal

We added 10 mL/g resin Solution D and shook this for 5 min (Figure 5c). Solution D was drained and washed once with NMP. This process was repeated twice, washing with NMP (5 mL/g resin) five times. Finally, a mini-cleavage was performed to assess the complete removal of the *o*-NBS group (Note 3) (Figure 6C).

Note 3: To evaluate the *o*-NBS removal step, a mini-cleavage should be performed to assess complete removal of the *o*-NBS group from the methylated amino acid. In the case of observing amino acid with *o*-NBS group, repeat steps (Section 2.4.7). On the other hand, if complete removal of *o*-NBS is achieved, continue to Section 2.4.8.

#### 2.4.8. Fmoc Protection

To protect the free amino group with Fmoc, 5 mL/g resin of Solution E were added into the reactor and 1 Eq of DIEA was added immediately (Figure 5d), and then this was shaken for 3 h. The Chloranil test (Note 4) or mini-cleavage were conducted to assess the complete Fmoc group protection (Figure 6D).

Note 4: To evaluate the Fmoc protection step, the chloranil test can be performed to determine the presence of free secondary amines. The results can be yellow resin: complete protection and absence of free secondary amines (continue with Section 2.4.9), or blue-green resin: incomplete protection and presence of free secondary amines (repeat steps (Section 2.4.8)). However, mini-cleavage is recommended to assess the complete protection of the Fmoc group on the free amino group of the methylated amino acid. In case of observing an amino acid without an Fmoc group, repeat steps (Section 2.4.8) again. In case of complete protection with Fmoc, continue with cleavage Section 2.4.9.

#### 2.4.9. Cleavage and Work-Up

After completing the previous steps, the resin was washed twice with DMF and twice with DCM (5 mL/g resin). Cleavage of the methylated and Fmoc-protected amino acid was performed using a cleavage solution of 1% TFA in DCM. The cleavage solution was added to the resin (10 mL/g resin), the mixture was shaken for 1 min, and then drained over 30 mL of MilliQ water. This step was important to preventing the cleavage of the side chain protecting groups due to the high acid concentration. The cleavage and the draining step were repeated four times. The DCM from the obtained mixture was evaporated, leaving behind the precipitated amino acid in the MilliQ water. Then, 10 mL of ACN were added to dissolve the precipitated amino acid. The solution was transferred into Falcon tubes, frozen using liquid nitrogen, and finally lyophilized for 48 h.

### 2.5. Characterization

Fmoc-N-Me-AA-OH was characterized by high-performance liquid chromatography (HPLC) to assess purity and by LC-MS to confirm its identity. Additionally, high-resolution mass spectrometry (HRMS) and nuclear magnetic resonance (NMR) spectroscopy (1H NMR, 13C NMR, and DEPT 135) were employed to determine the molecular structure and chemical composition of the samples. These characterization methods allowed for the evaluation of the quality and properties of the synthesized product.

#### 2.5.1. Reverse-Phase High-Performance Liquid Chromatography (RP-HPLC)

The determination of the percentage of purity of the Fmoc-N-Me-AA-OH was performed by RP-HPLC using a 30 to 100% acetonitrile (ACN) gradient in 8 min for Fmoc-N-Me-βAla-OH and 50 to 100% acetonitrile gradient in 8 min for Fmoc-N-Me-Thr(tBu)-OH. Column: C18 XBridge BEH 130, 3.5 μm, 4.5 mm × 100 mm. Solvents: Water with 0.045% of TFA and ACN with 0.036% of TFA. Wavelength Detection: 220 nm. Flow: 1 mL/min.

#### 2.5.2. Liquid Chromatography-Mass Spectrometry (LC-MS)

The presence of Fmoc-N-Me-AA-OH was evaluated by LC-MS, which showed the peaks for the corresponding [M+H]1+ of each AA, using a 30 to 100% ACN gradient in 3.5 min for Fmoc-N-Me-βAla-OH and 50 to 100% acetonitrile gradient in 3.5 min for Fmoc-N-Me-Thr(tBu)-OH. Column: XSelect 3.5 μm, 4.6 × 50 mm. Solvent: water 0.1% formic acid and ACN 0.07% formic acid. Wavelength Detection: 220 nm. Flow: 1.6 mL/min.

#### 2.5.3. Electrospray Ionization Time-of-Flight (ESI-TOF) HR-MS

The ESI-TOF analysis of each amino acid was carried out on a LC/MSD-TOF Agilent Technologies G1969A TOF Mass Spectrometer System, using eluent: H_2_O:CH_3_CN (1:1) 200 μL/min, at 175.0 V.

#### 2.5.4. Nuclear Magnetic Resonance (NMR)

The amino acids were characterized by NMR, ^1^H, ^13^C, and DEP 135.

### 2.6. Reuse of 2-CTC Resin

**Figure 7:**
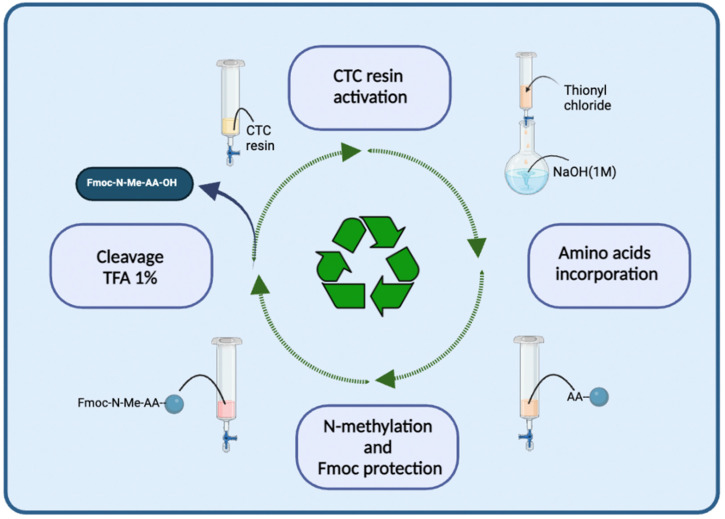
Schematic diagram of the steps to obtain Fmoc-N-Me-AA-OH in the reuse cycle of the 2-CTC resin.

Promoting the use of 2-CTC resin as a temporary protector in the Fmoc-N-Me-AA-OH synthesis process could help to reduce process costs due to the reuse of the resin (Figure 7). After cleavage and work-up (Section 2.4.9), the resin was washed with DCM five times (5 mL/g resin) in the same reactor, and then we continued with the synthetic chemistry (Section 2.4).

## 3. Results and Discussion

### 3.1. Yield and Purity

In this work, the synthesis of two Fmoc-N-Me-AA-OH from two methylation strategies, using dimethyl sulfate and methyl iodide, is presented. Table 1 shows a summary of the information collected for the Fmoc-N-Me-Thr(tBu)-OH and Fmoc-N-Me-βAla-OH synthesized. The theoretical yield was calculated according to Equation (2) and the % purity was evaluated by integrating the main peak in the corresponding chromatogram (Table 2).

(2) Theoretical yield=Resin loading×mg resin×Molecular weight AA

As can be seen, two methylated amino acids were obtained, Fmoc-N-Me-Thr(tBu)-OH and Fmoc-N-Me-βAla-OH, one being an alpha amino acid and the other a beta amino ac-id. Both amino acids were synthesized under the same conditions, resulting in a quite similar yield, which exceeded 70%. Regarding the methylation process, it was necessary to perform multiple reaction cycles to achieve complete methylation in the case of Thr. This could be attributed to its hindrance. This process was easily monitored by HPLC, as the two species have different retention times (approximately a 1 min difference under working conditions).

The yields obtained with the two methylating reagents were similar, with a variation between 1% and 10% for both amino acids. Additionally, a purity greater than 90% was obtained for both amino acids, demonstrating the great effectiveness of both reagents in the methylation stage (Table 1).

### 3.2. Characterization

**Figure 8:**
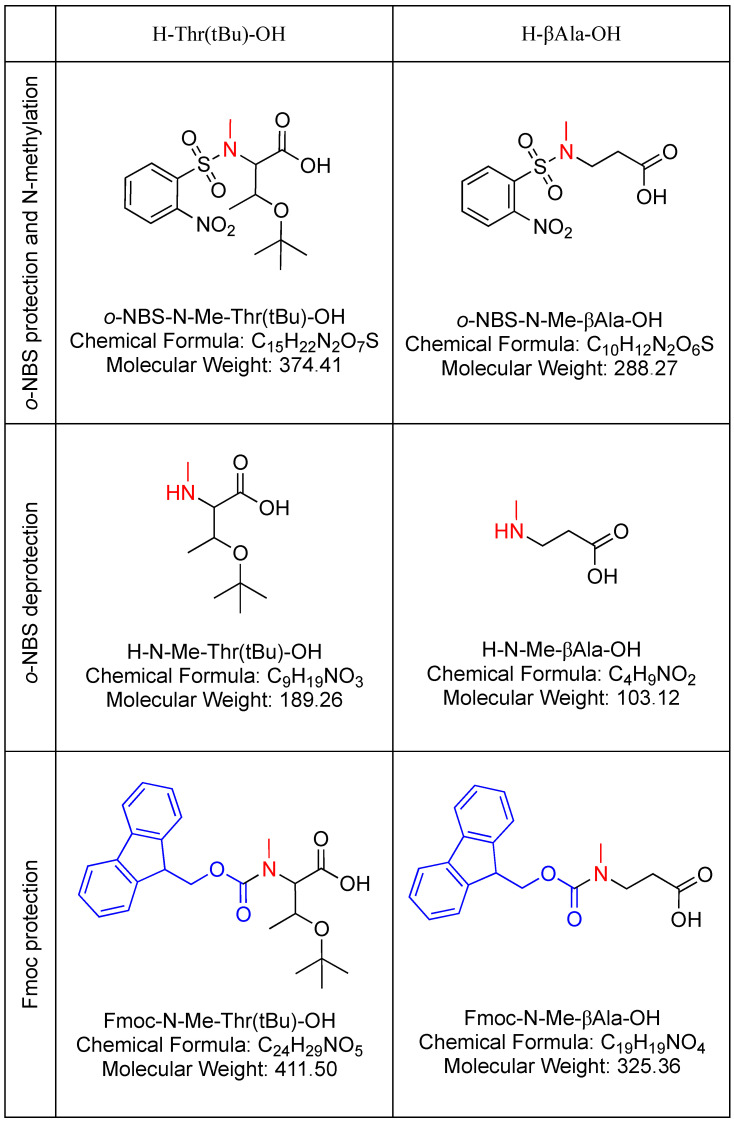
F structure of each molecule obtained after mini-cleavage and mass spectra analysis at each methylation step.

As shown in Figure 8 and Table 3, after each methylation step (*o*-NBS protection and N-methylation, *o*-NBS deprotection, and Fmoc protection), both amino acids were correctly identified based on [M+H]1+. The results of the ESI/MS are shown in Tables S1 and S2.

The results obtained by ESI-TOF showed HR-MS Fmoc-N-Me-βAla-OH; calculated mass for (C_19_H_19_NO_4_) [M+H]+1 = 325.3640. Mass found: [M+H]+1 = 326.1393, Abund 96.13; [M+H+Na]+1 = 348.1209, Abund 89.88; [M+H+K]+1 = 364.0911 (see Figures S1 and S2 in Supplementary Materials). The impurities associated with Fmoc-N-Me-βAla-OH were not impurities with a carboxylic end and, therefore, they did not interfere with the peptide synthesis process.

The HR-MS Fmoc-N-Me-Thr(tBu)-OH, calculated mass for (C_24_H_29_NO_5_) [M+H]+1 = 411.4980. Mass found: [M+H]+1 = 412.2118, Abund Match 81.04; (C_24_H_29_NNaO_5_) [M+H+Na]+1 = 434.1937 Abund Match 88.79; (C_24_H_29_KNO_5_) [M+H]+1 = 450.1661 Abund Match 83.53; and (C_20_H_22_NO_5_) [M+H]+1 = 356.1492, Abund Match 95.29. This shows a partial deprotection of the tBu protector of the threonine side chain, which is also evident in the ^1^HNMR spectrum, indicating the presence of Fmoc-N-Me-Thr-OH (Figure S3 and S4). Its occurrence may be attributed to the cleavage step resulting in the removal of the tBu group or due to the hindrance of the secondary alcohol; however, its presence did not interfere in the purity of the final peptide.

Also, through the use of NMR, we obtained that for ^1^H NMR Fmoc-N-Me-βAla-OH 400 Mhz, DMSO (d6): 2.32 ppm, t(2H); 2.79 ppm, s(1H); 3.34 ppm, t(2H); 4.27 ppm, t(1H); 4.34 ppm, d(2H); 7.33 ppm, t(2H); 7.41 ppm, t(2H); 7.63 ppm, d(2H); 7.88 ppm, t(2H); 12.25 ppm, s(1H). ^13^C NMR Fmoc-N-Me-βAla-OH, 400 MHz; CDCl_3_: 32.60 ppm; 35.21 ppm; 44.92 ppm; 47.33 ppm; 67.35 ppm; 120.00 ppm; 124.72 ppm; 125.00 ppm; 127.10 ppm; 127.72 ppm; 141.39 ppm; 143.98 ppm; 156.24 ppm; 176.71 ppm. (see Figure S5–S8 in Supplementary Materials).

^1^H NMR Fmoc-N-Me-Thr(tBu)-OH, 400 MHz DMSO (d6): 1.06 ppm, d (3H); 1.10 ppm, s, 11(H); 2.92 ppm, s, (1H); 2.97 ppm, s, (3H); 4.30 ppm, m, (3H); 4.4 ppm, m, (1H); 4.5 ppm, d (1H); 7.33 ppm, m, (2H); 7.42 ppm, m, (2H); 7.65 ppm, m, (2H); 7.9 ppm, m, (2H); 12.78 pp, s (1H). ^13^C NMR Fmoc-N-Me-Thr(tBu)-OH, 400 MHz CDCl3: 20.05 ppm; 28.38 ppm; 34.41 ppm; 47.23 ppm; 64.29 ppm; 67.66 pmm; 68.15 ppm; 75.87 ppm; 120.01 ppm; 124.79 ppm; 125.01 ppm; 127.12 ppm; 127.80 ppm; 141.40 ppm; 143.63 ppm; 143.84 ppm; 156.58 ppm; 157.73 pmm; 172.74 ppm (see Figures S11 and S15 in Supplementary Materials).

In the proton spectrum, the partial deprotection of the tBu group of the hydroxyl of the beta carbon is also evident, making it possible to calculate the % deprotection from two very characteristic signals of the spectrFum: the methyl group of the beta carbon and the methyl bonded to nitrogen.

Considering the relationship between the methyl protons attached to the beta carbon, we can calculate the percentage of deprotection of the tBu group as (2.62 + 1.29 = 3.91) (1.29/3.91 = 0.3299 × 100 = 32.99%) unprotected.

If we take as reference the other characteristic signal, the methyl protons linked to nitrogen, we have (2.59 + 1.23 = 3.82) (1.23/3.82 = 0.3219 × 100 = 32.19%).

In the N-methylation of Fmoc-N-Me-Thr(tBu)-OH that has two chiral centers (the alpha carbon and the beta carbon), the proton spectrum at 400 MHz indicates a single signal for the proton attached to the carbon alpha, (see Figure S10 in Supplementary Materials), which indicates to us that we are in the presence of a single stereoisomer: Fmoc-L-N-Me-Thr(tBu)-OH.

### 3.3. Recycling and Reagent Usage

To reduce 2-CTC resin costs, this protocol included steps allowing for the reuse of the 2-CTC resin (Figure 7). The 2-CTC resin served as a temporary protecting group for the carboxylic end of the amino acids, facilitating the methylation and subsequent Fmoc protection at the amino end. Afterward, the amino acid was cleaved from the 2-CTC resin, resulting in the synthesis of Fmoc-N-Me-βAla-OH and Fmoc-N-Me-Thr(tBu)-OH.

During the resin reuse process (Section 2.6), the loading values obtained were compared between the first and second activation of the resin. The results demonstrate that the resin maintained its properties allowing for a new synthesis cycle of Fmoc-N-Me-βAla-OH and Fmoc-N-Me-Thr(tBu)-OH. The obtained products had a purity above 90% in all cases (Table 1). Even though the yield was slightly higher in the first cycle of resin activation, yields above 70% were achieved in the second cycle, highlighting the feasibility of resin reuse.

This reuse strategy reduced the consumption of 2-CTC resin by approximately 80% for each preparation of Fmoc-N-Me-AA-OH.

Additionally, with the two methylation reagents used, very similar results were obtained, both in terms of yield and in purity, for the two cycles of resin use.

Dissolution methods such as saponification, which is carried out under reflux conditions in ethyl acetate in the presence of lithium iodide for 16 h [12], turn out to be much more labor-intensive. However, this method enables us to rapidly obtain a significant amount, approximately between 1 and 2 millimoles of Fmoc-N-Me-AA-OH, without racemization.

## 4. Conclusions

A protocol is presented for the synthesis of two Fmoc-N-Me-AA-OH, using the methylation strategy based on the Biron−Kessler method in solid-phase with 2-CTC resin as a temporary protector and using *o*-NBS to protect the α-amino groups. The subsequent alkylation step was carried out using two strategies in parallel to compare the use of the most common reagents in peptide synthesis, aiming to assess the effectiveness of each. This method enables the preparation of a substantial quantity of these protected amino acids within a few hours, without racemization.

## Figures and Tables

**Table 1:** Synthesized crude Fmoc-N-Me-AA-OH and its characterization after the first activation of 2-CTC resin and after the second activation with reused 2-CTC resin.

*o*-NBS Deprotection Reagents	Fmoc-N-Me-[AA]-OH	Resin (mg)	Loading (mEq/g)	MW (g/mol)	Theoretical Yield (mg)	Experimental Yield (mg)	% Yield	Retention Time (tr)	% Purity
	First activation of 2-CTC resin								
Dimethyl sulfate	**1.** [βAla]	160.2	1.76	325.4	91.9	85.3	92.8	5.253	98.59
	**3.** [Thr(tBu)]	152.5	1.54	411.5	97.0	71.1	73.3	6.007	90.83
Methyl iodide	**2.** [βAla]	155.5	1.76	325.4	89.2	81.5	91.3	5.257	94.07
	**4.** [Thr(tBu)]	155.5	1.54	411.5	98.9	83.7	84.6	6.050	93.29
	REUSE: Second activation of 2-CTC resin								
Dimethyl sulfate	R1. [βAla]	150.8	1.773	325.4	87.1	74.0	84.9	5.220	98.38
	R3. [Thr(tBu)]	111.0	1.36	411.5	62.3	50.5	81.0	6.014	97.15
Methyl iodide	R2. [βAla]	141.3	1.816	325.4	83.5	67.7	81.1	5.238	99.2
	R4. [Thr(tBu)]	124.5	1.42	411.5	73.0	59.9	82.0	6.020	94.63

**Table 2:** Analysis by reverse phase high performances liquid chromatography (RP-HPLC) of methylated crude amino acid for the two methylation reagents and after the first and second activation of reused 2-CTC resin, using a 30 to 100% acetonitrile gradient in 8 min for Fmoc-N-Me-βAla-OH and 50 to 100% acetonitrile gradient in 8 min for Fmoc-N-Me-Thr(tBu)-OH.

		Fmoc-N-Me-βAla-OH	Fmoc-N-Me-Thr(tBu)-OH
**Synthesis with first activation of 2-CTC-resin**	**Dimethyl sulfate**	1	3
		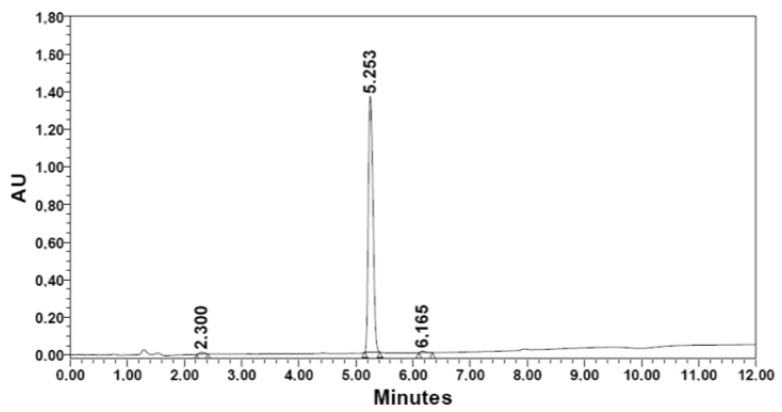	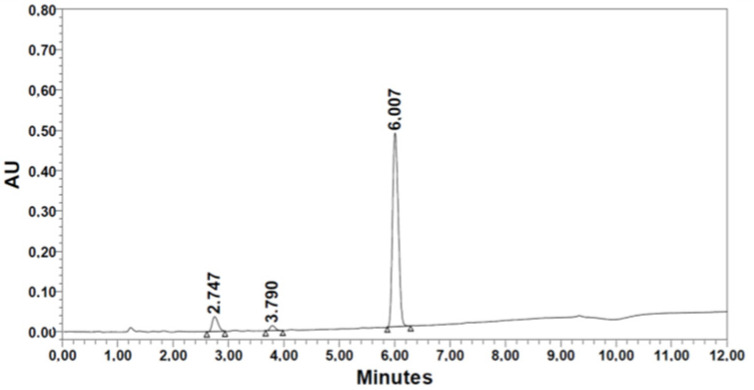
	**Methyl iodide**	2	4
		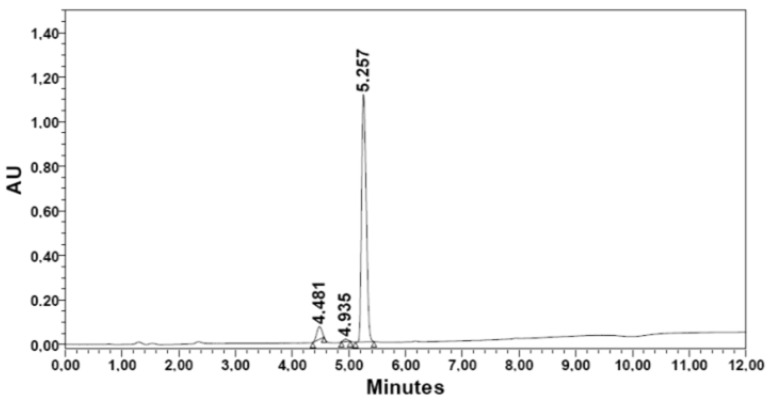	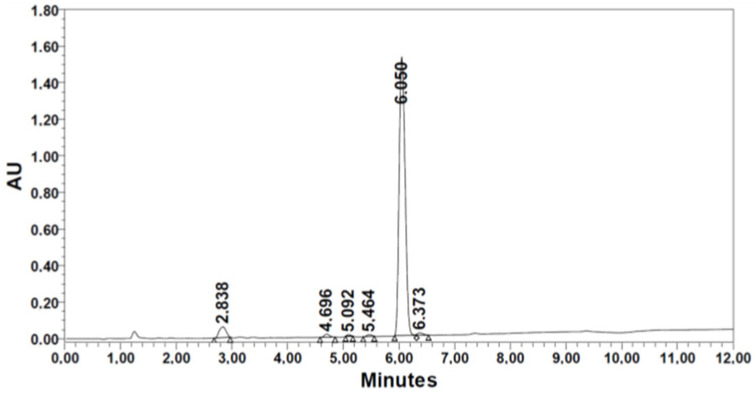
**REUSE: Synthesis with second activation of 2-CTC-resin**	**Dimethyl sulfate**	R1	R3
		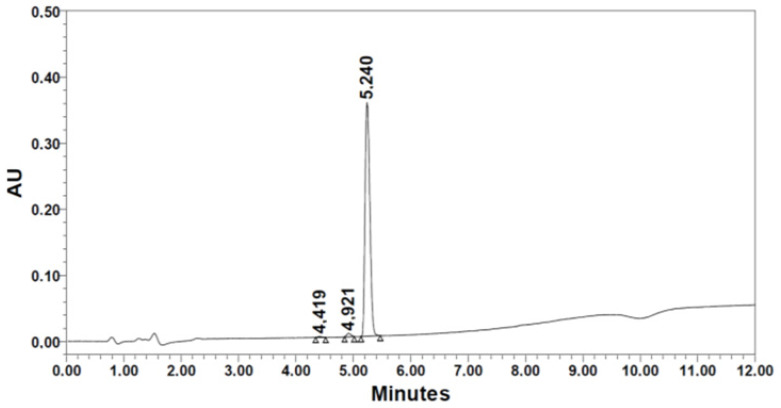	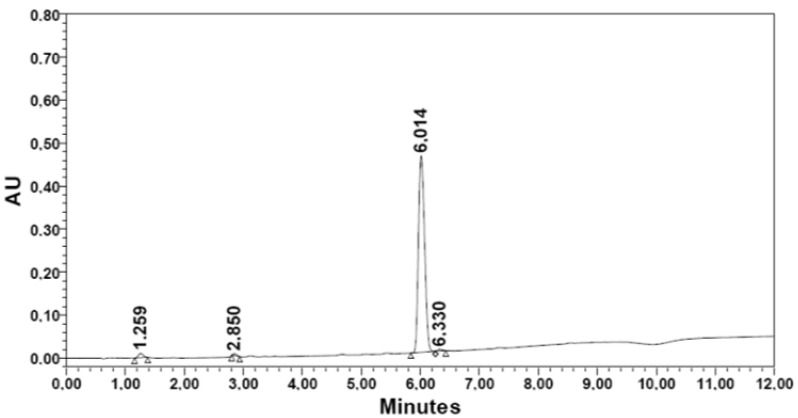
	**Methyl iodide**	R2	R4
		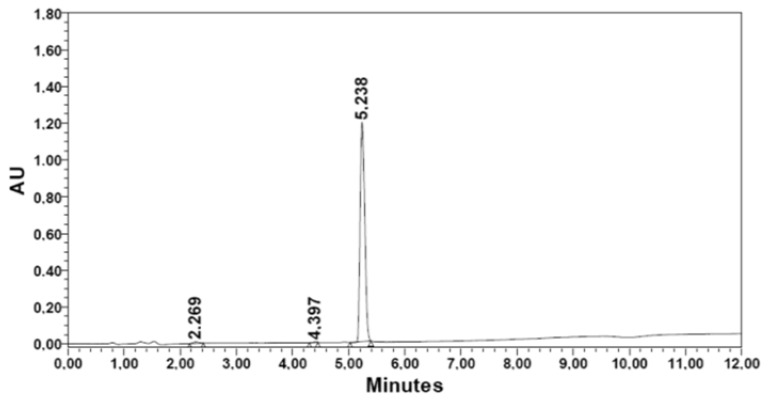	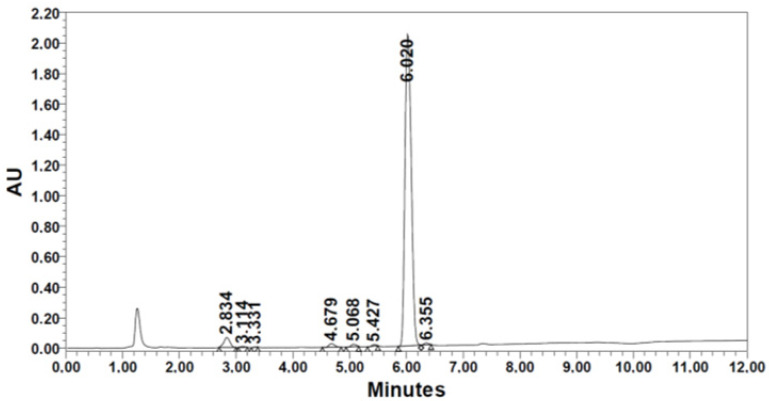

**Table 3:** Characterization of mini-cleavage products after each step of methylation: *o*-NBS protection and N-methylation; *o*-NBS deprotection; Fmoc protection.

Reagents to Remove o-NBS	Methylation Steps	Sequence	MW (g/mol)	Ion M1+		
				Theoretical	Experimental (First Activation)	Experimental (Second Activation)
Dimethyl sulfate	*o*-NBS protection and N-methylation	*o*-NBS-N-Me-βAla-OH	288.27	289.27	*	289.3
		*o*-NBS-N-Me-Thr(tBu)-OH	374.41	375.41	*	375.4
	*o*-NBS deprotection	H-N-Me-βAla-OH	103.12	104.12	104.2	104.2
		H-N-Me-Thr(tBu)-OH	189.26	190.26	190.3	190.4
	Fmoc protection	Fmoc-N-Me-βAla-OH	325.36	326.36	326.5	326.5
		Fmoc-N-Me-Thr(tBu)-OH	411.5	412.5	412.5	412.4
Methyl iodide	*o*-NBS protection and N-methylation	*o*-NBS-N-Me-βAla-OH	288.27	289.27	*	289.3
		*o*-NBS-N-Me-Thr(tBu)-OH	374.41	375.41	*	375.4
	*o*-NBS deprotection	H-N-Me-βAla-OH	103.12	104.12	104.2	104.2
		H-N-Me-Thr(tBu)-OH	189.26	190.26	190.3	190.4
	Fmoc protection	Fmoc-N-Me-βAla-OH	325.36	326.36	326.5	326.4
		Fmoc-N-Me-Thr(tBu)-OH	411.5	412.5	412.5	412.5

* The completion of the reaction with o-NBS-Cl was determined by conducting the ninhydrin test to detect the presence of free amines.

## Data Availability

Data sharing not applicable. No new data were created or analyzed in this study. Data sharing is not applicable to this article.

## Supplementary Materials

The following supporting information can be downloaded at: https://www.mdpi.com/article/10.3390/mps6060110/s1, Table S1: ESI-MS analysis of Fmoc-N-Me-βAla-OH and Fmoc-N-Me-Thr(tBu)-OH with first activation of CTC resin using dimethyl sulfate and iodomethane, both as N-methylation reagents; Table S2: ESI-MS analysis of Fmoc-N-Me-βAla-OH and Fmoc-N-Me-Thr(tBu)-OH with second activation of CTC resin using dimethyl sulfate and iodomethane, both as N-methylation reagents; Figure S1: ESI-TOF analysis of Fmoc-N-Me-βAla-OH using dimethyl sulfate as N-methylation reagent; Figure S2: ESI-TOF analysis of Fmoc-N-Me-βAla-OH using iodomethane as N-methylation reagent; Figure S3: ESI-TOF analysis of Fmoc-N-Me-Thr(tBu)-OH using dimethyl sulfate as N-methylation reagent; Figure S4: ESI-TOF analysis of Fmoc-N-Me-Thr(tBu)-OH using iodomethane as N-methylation reagent; Figure S5. NMR ^1^H spectrum of Fmoc-N-Me-βAla-OH at 400 MHz in DMSO(d6); Figure S6. NMR ^1^H spectrum of Fmoc-N-Me-βAla-OH at 400 MHz in CDCl_3_; Figure S7. NMR DEP 135 spectrum of Fmoc-N-Me-βAla-OH at 400 MHz in CDCl_3_; Figure S8. Overlaid ^13^C and DEP 135 spectra of Fmoc-N-Me-βAla-OH at 400 MHz in CDCl_3_; Figure S9. NMR ^1^H spectrum of Fmoc-N-Me-Thr(tBu)-OH at 400 MHz in DMSO(d6); Figure S10. NMR ^1^H spectrum of Fmoc-NMe-Thr(tBu)-OH enlarged aliphatic zone; Figure S11. NMR ^13^C spectrum of Fmoc-N-Me-Thr(tBu)-OH at 400 MHz in CDCl_3_; Figure S12. NMR DEP 135 spectrum of Fmoc-N-Me-Thr(tBu)-OH at 400 MHz in CDCl_3_; Figure S13. Overlaid ^13^C and DEP 135 spectra of Fmoc-N-Me-Thr(tBu)-OH at 400 MHz in CDCl_3_.
