# Supplementary material for: Protocol for Facile Synthesis of Fmoc-N-Me-AA-OH Using 2-CTC Resin as Temporary and Reusable Protecting Group
Source: Methods Protoc. 2023 Nov 13;6(6):110. doi: 10.3390/mps6060110 (PMC10660853; doi:10.3390/mps6060110)
Supplement: Supplementary file 1 [file mps-06-00110-s001.zip › mps-2633335-supplementary.pdf]

Supplementary Information

Facile Synthesis of Fmoc-N-Me-AA-OH using CTC resin as Temporary Protecting Group

Tanya Román 1,2,3, Gerardo Acosta 3,4, Constanza Cárdenas 1, Beatriz G. de la Torre 5, Fanny Guzmán 1,* and Fernando Albericio 3,4,6,*

1. Núcleo Biotecnología Curauma, Pontificia Universidad Católica de Valparaíso, Valparaíso 2373223, Chile; tanya.roman.21@gmail.com (T.R.); constanza.cardenas@pucv.cl (C.C.)

2. Doctorado en Biotecnología, Pontificia Universidad Católica de Valparaíso, Universidad Técnica Federico Santa María, Valparaíso 2373223, Chile

3. Department of Organic Chemistry and CIBER-BBN, Networking Centre on Bioengineering, Biomaterials and Nanomedicine, University of Barcelona, 08028 Barcelona, Spain; gerardoacosta@ub.edu

4. Institute for Advanced Chemistry of Catalonia (IQAC-CSIC), Jordi Girona 18-26, 08034 Barcelona, Spain

5. KwaZulu-Natal Research Innovation and Sequencing Platform (KRISP), School of Laboratory Medicine and Medical Sciences, College of Health Sciences, University of KwaZulu-Natal, Durban 4041, South Africa; garciadelatorreb@ukzn.ac.za; (B.G.d.l.T)

6. School of Chemistry and Physics, University of KwaZulu-Natal, Durban 4001, South Africa

*Correspondence: fanny.guzman@pucv.cl (F.G.); albericio@ub.edu (F.A.)

Table S1: ESI-MS analysis of Fmoc-N-Me-βAla-OH and Fmoc-N-Me-Thr(tBu)-OH with first activation of CTC resin using dimethyl sulfate and iodomethane, both as N-methylation reagents. A) Analysis of mini-cleavage after *o*-NBS deprotection. B) Analysis of mini-cleavage after Fmoc protection.

Fmoc-N-Me-AA-OH synthesis with first activation of CTC resin		
	Dimethyl sulfate	Iodomethane
A) o-NBS deprotection	1-H-N-Me-βAla-OH	2-H-N-Me-βAla-OH
	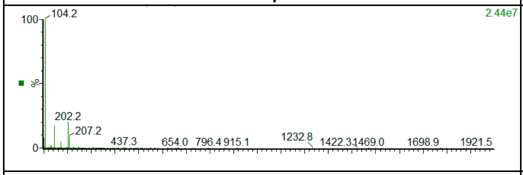	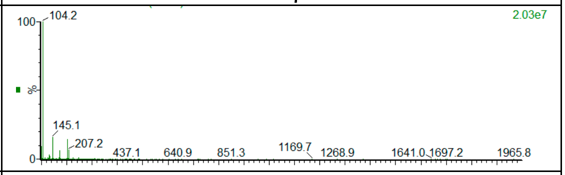
	3-H-N-Me-Thr(tBu)-OH	4-H-N-Me-Thr(tBu)-OH
	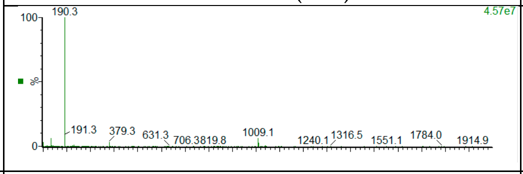	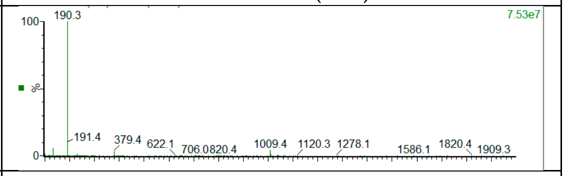
B) Fmoc protection	1-Fmoc-N-Me-βAla-OH	2-Fmoc-N-Me-βAla-OH
	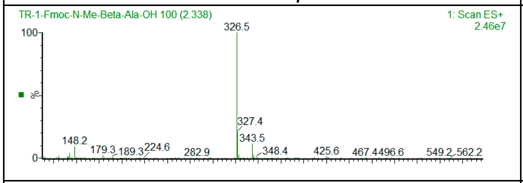	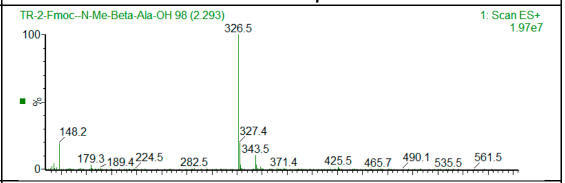
	3-Fmoc-N-Me-Thr(tBu)-OH	4-Fmoc-N-Me-Thr(tBu)-OH
	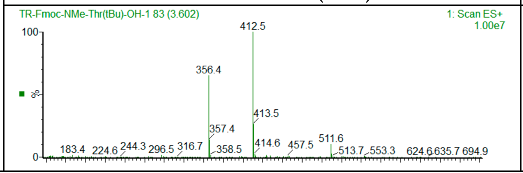	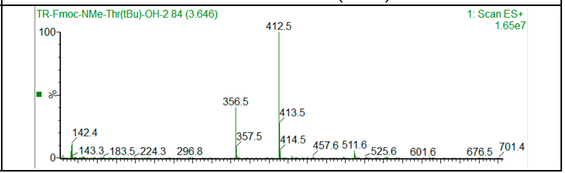

Table S2: ESI-MS analysis of Fmoc-N-Me-βAla-OH and Fmoc-N-Me-Thr(tBu)-OH with second activation of CTC resin using dimethyl sulfate and iodomethane, both as N-methylation reagents. A) Analysis of mini-cleavage after *o*-NBS protection and N-methylation. B) Analysis of mini-cleavage after *o*-NBS deprotection. C) Analysis of mini-cleavage after Fmoc protection.

<table>
<tr><th colspan="3">Fmoc-N-Me-AA-OH synthesis with second activation of CTC resin</th></tr>
<tr><td></td><td>Dimethyl sulfate</td><td>Iodomethane</td></tr>
<tr><td rowspan="4">A) o-NBS protection and N-methylation</td><td>R1-Ns-N-Me-βAla-OH</td><td>R2-Ns-N-Me-βAla-OH</td></tr>
<tr><td>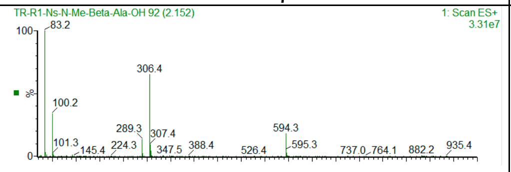
</td><td>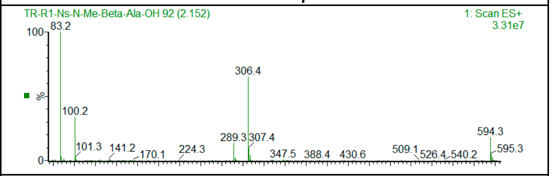
</td></tr>
<tr><td>R3-Ns-N-Me-Thr(tBu)-OH</td><td>R4-Ns-N-Me-Thr(tBu)-OH</td></tr>
<tr><td>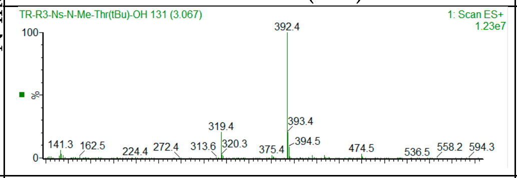
</td><td>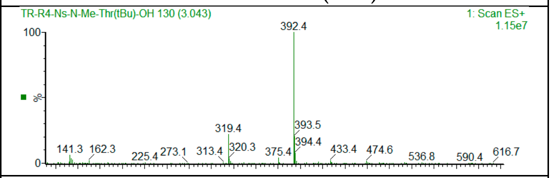
</td></tr>
<tr><td rowspan="4">B) o-NBS deprotection</td><td>R1-H-N-Me-βAla-OH</td><td>R2-H-N-Me-βAla-OH</td></tr>
<tr><td>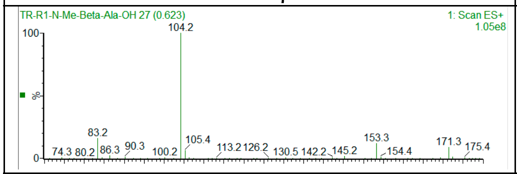
</td><td>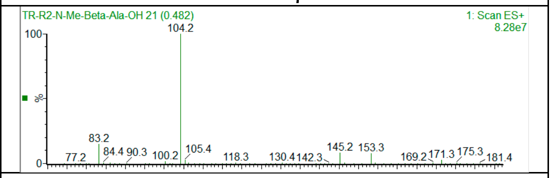
</td></tr>
<tr><td>R3-H-N-Me-Thr(tBu)-OH</td><td>R4-H-N-Me-Thr(tBu)-OH</td></tr>
<tr><td>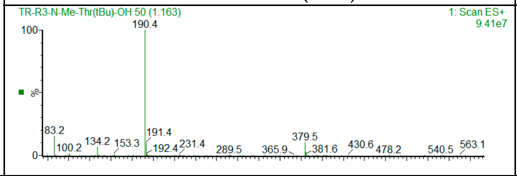
</td><td>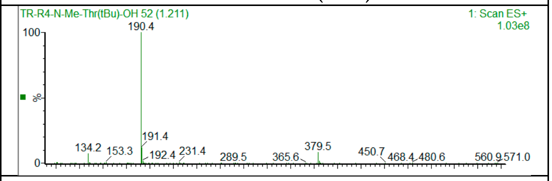
</td></tr>
<tr><td rowspan="4">C) Fmoc protection</td><td>R1-Fmoc-N-Me-βAla-OH</td><td>R2-Fmoc-N-Me-βAla-OH</td></tr>
<tr><td>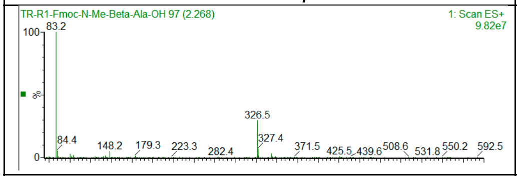
</td><td>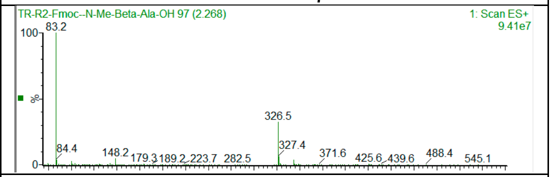
</td></tr>
<tr><td>R3-Fmoc-N-Me-Thr(tBu)-OH</td><td>R4-Fmoc-N-Me-Thr(tBu)-OH</td></tr>
<tr><td>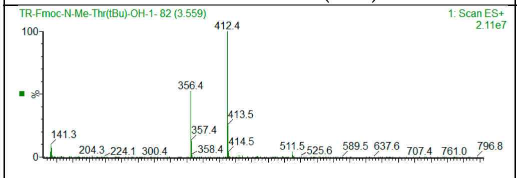
</td><td>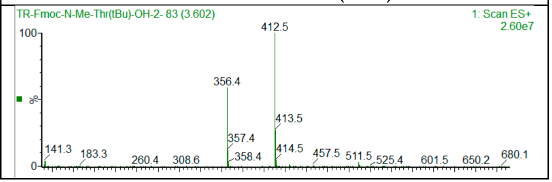
</td></tr>
</table>

R1-Fmoc-N-Me-βAla-OH

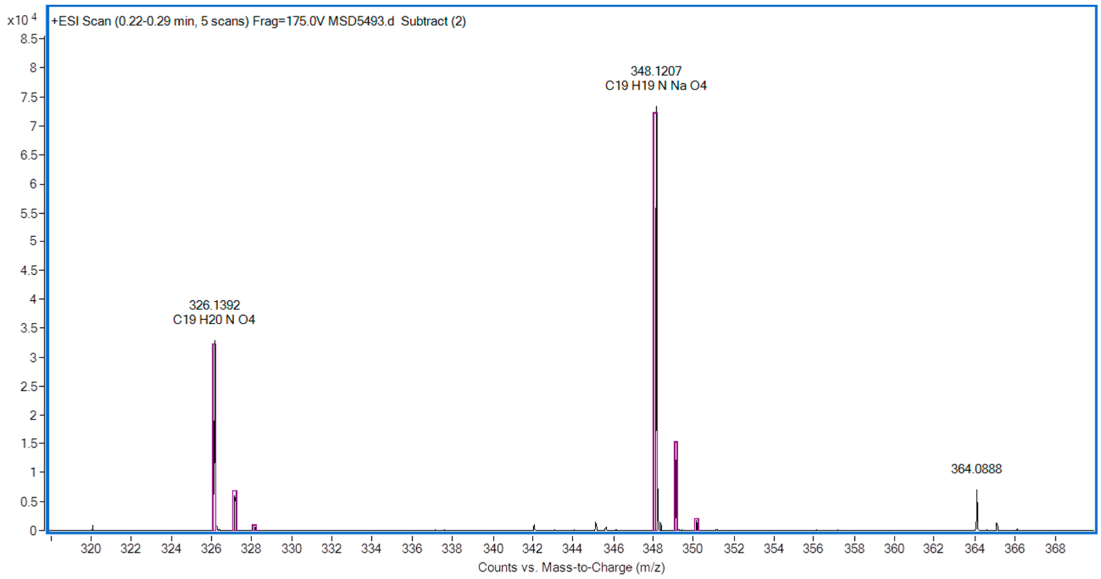

Figure S1: ESI-TOF analysis of Fmoc-N-Me-βAla-OH using dimethyl sulfate as N-methylation reagent.

R2-Fmoc-N-Me-βAla-OH

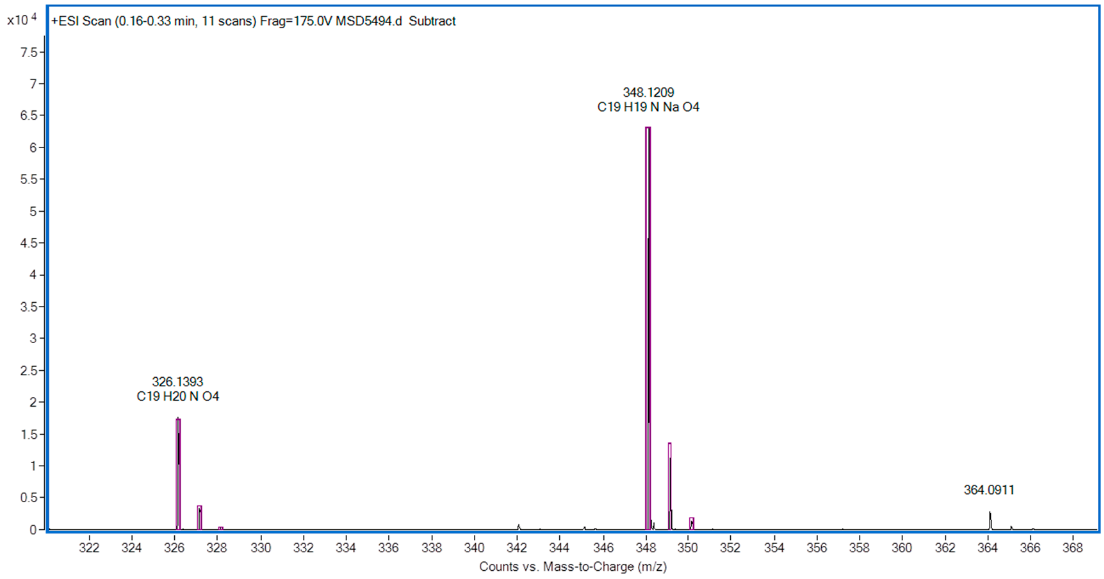

Figure S2: ESI-TOF analysis of Fmoc-N-Me-βAla-OH using iodomethane as N-methylation reagent.

R3-Fmoc-N-Me-Thr(tBu)-OH

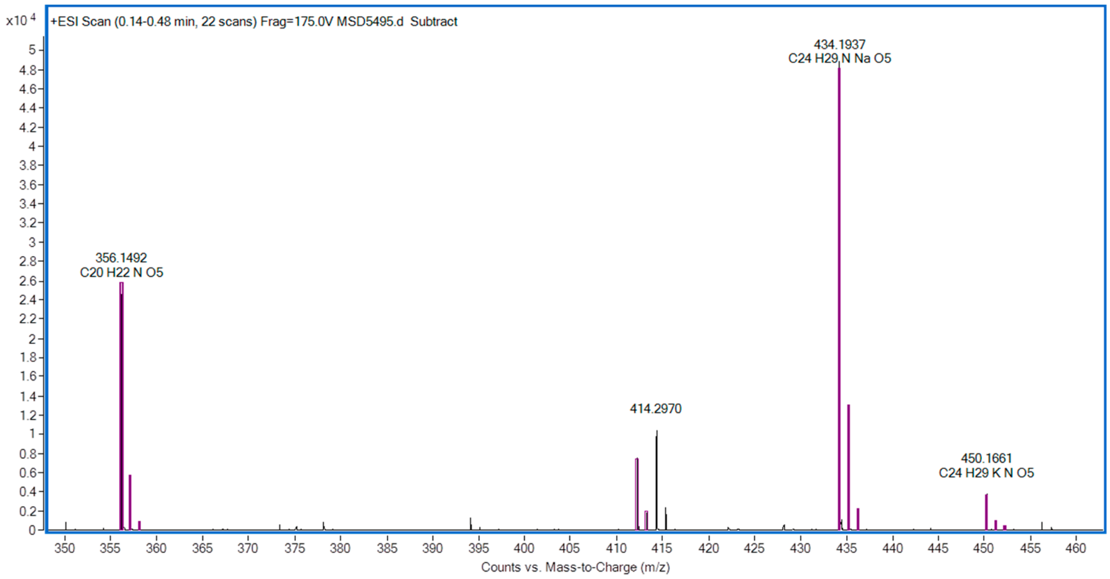

Figure S3: ESI-TOF analysis of Fmoc-N-Me-Thr(tBu)-OH using dimethyl sulfate as N-methylation reagent.

R4-Fmoc-N-Me-Thr(tBu)-OH

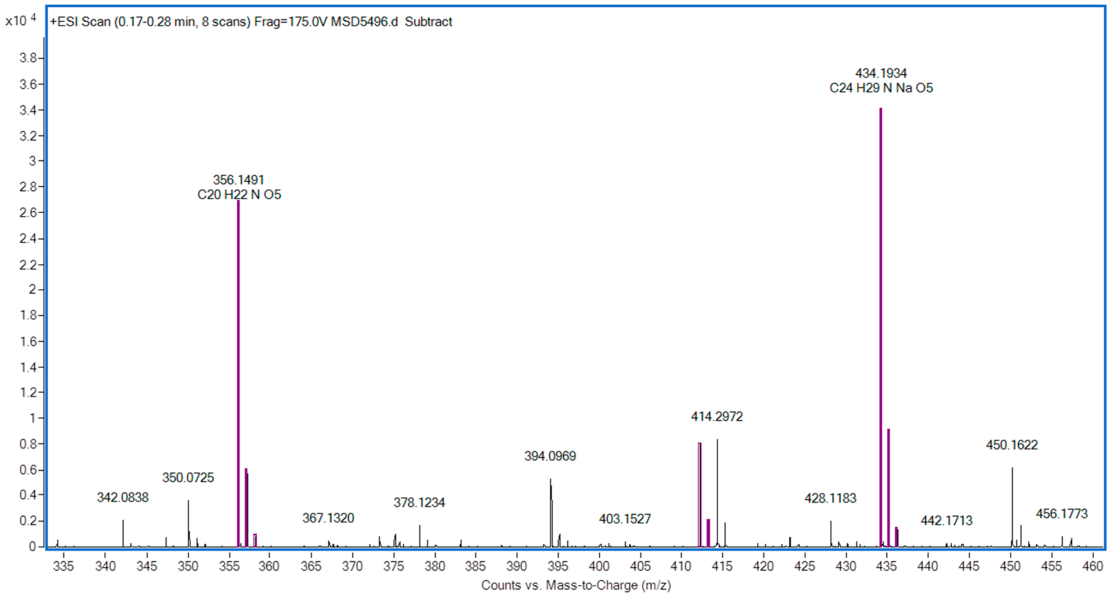

Figure S4: ESI-TOF analysis of Fmoc-N-Me-Thr(tBu)-OH using iodomethane as N-methylation reagent.

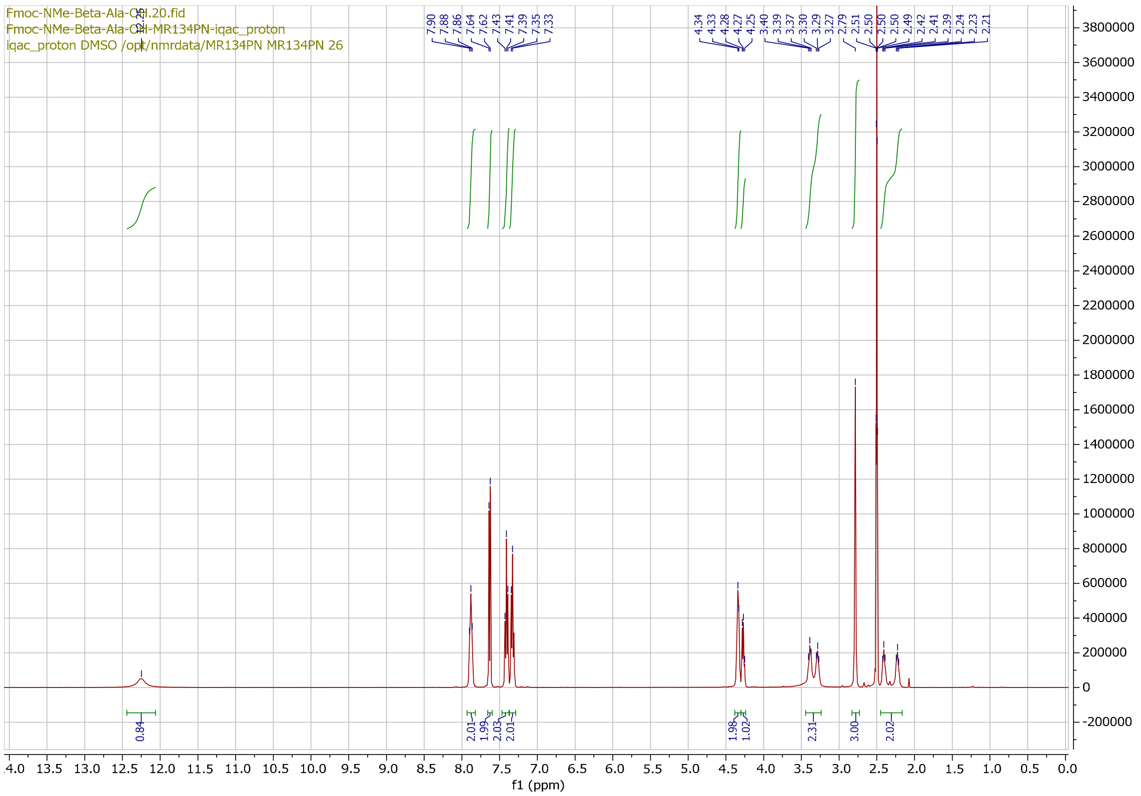

Figure S5: NMR ^{1}H spectrum of Fmoc-N-Me-βAla-OH at 400 MHz in DMSO(d6).

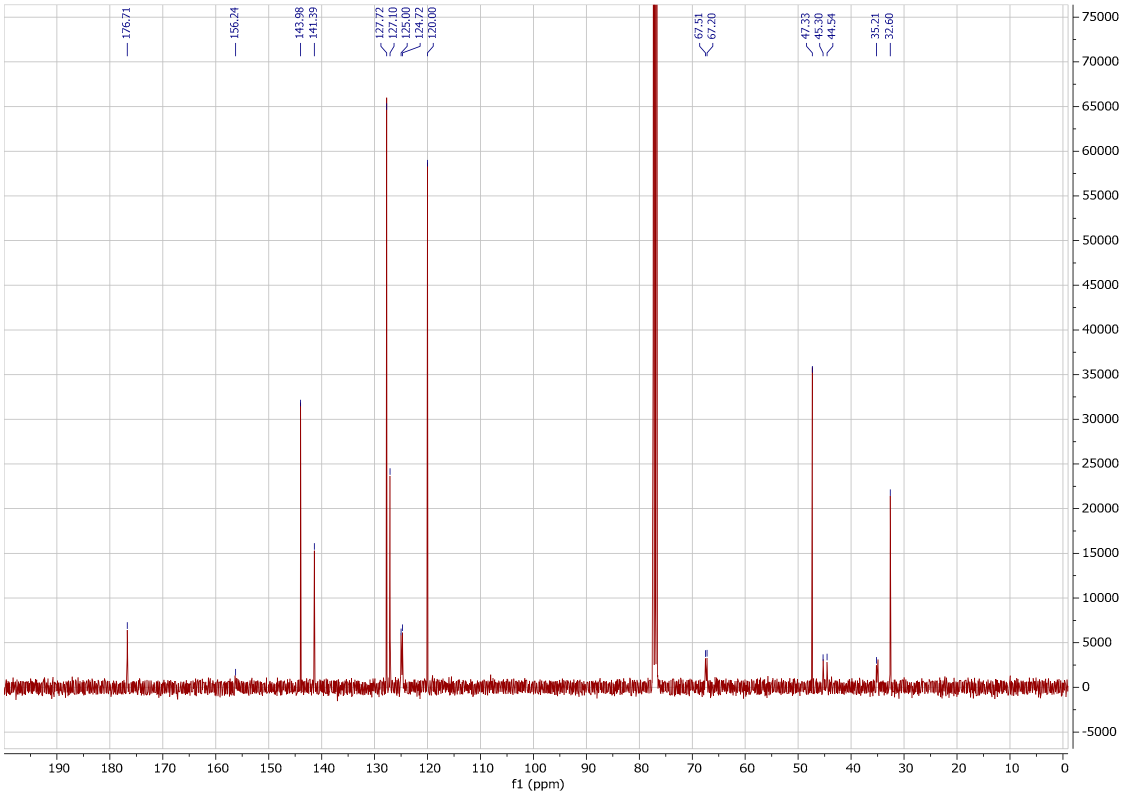

Figure S6: NMR ^{1}H spectrum of Fmoc-N-Me-βAla-OH at 400 MHz in $CDCl_3$.

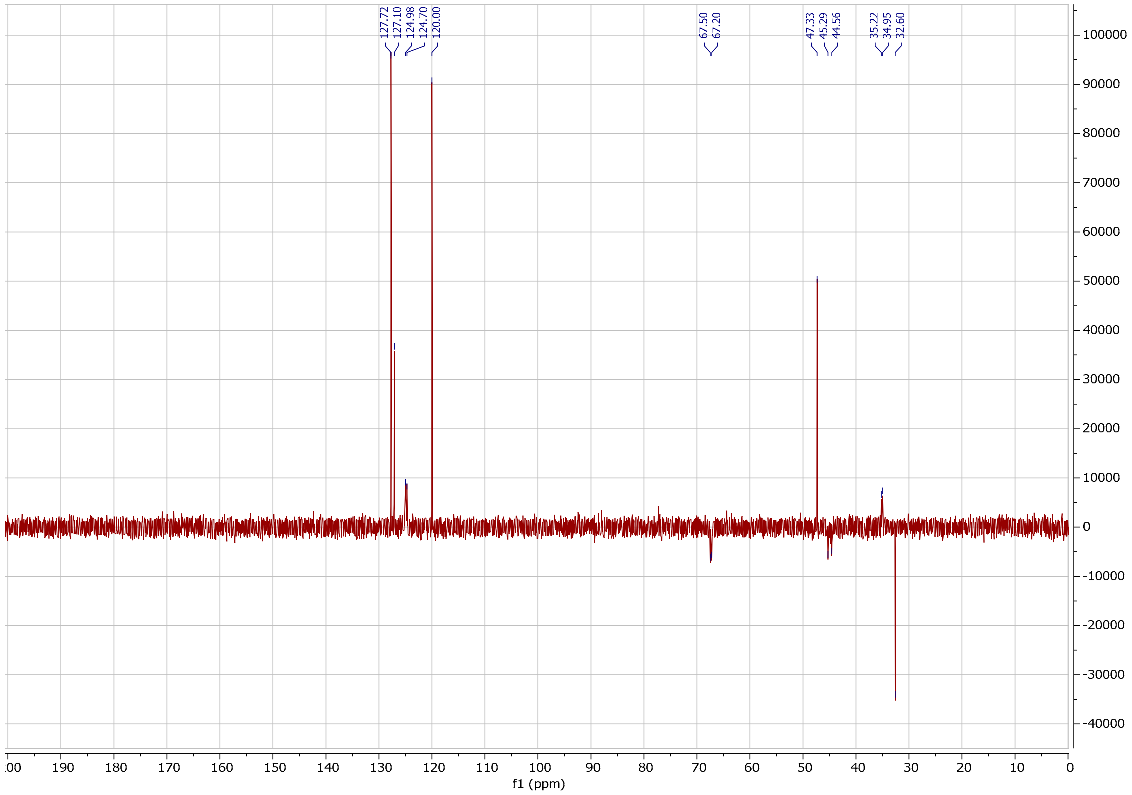

Figure S7: NMR DEP 135 spectrum of Fmoc-N-Me-βAla-OH at 400 MHz in $CDCl_3$.

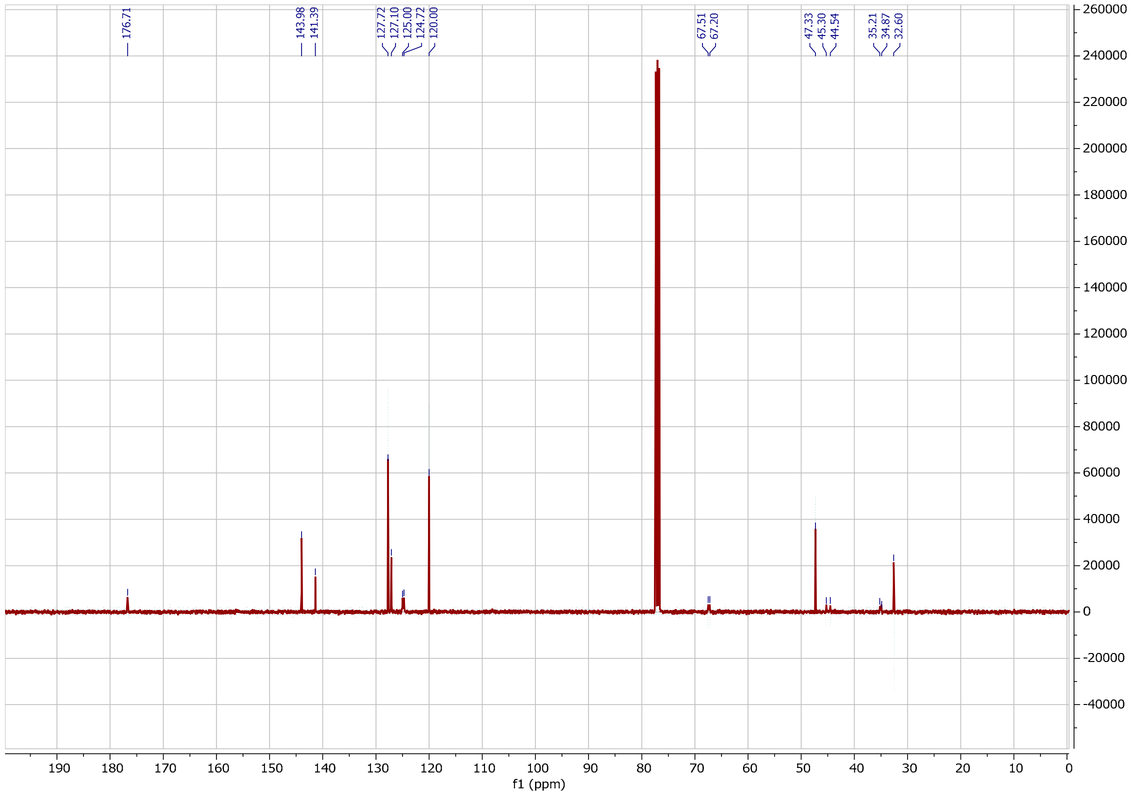

Figure S8: Overlaid ^{13}C and DEP 135 spectra of Fmoc-N-Me-βAla-OH at 400 MHz in $CDCl_3$.

Figure S9: NMR ^{1}H spectrum of Fmoc-N-Me-Thr(tBu)-OH at 400 MHz in DMSO(d6).

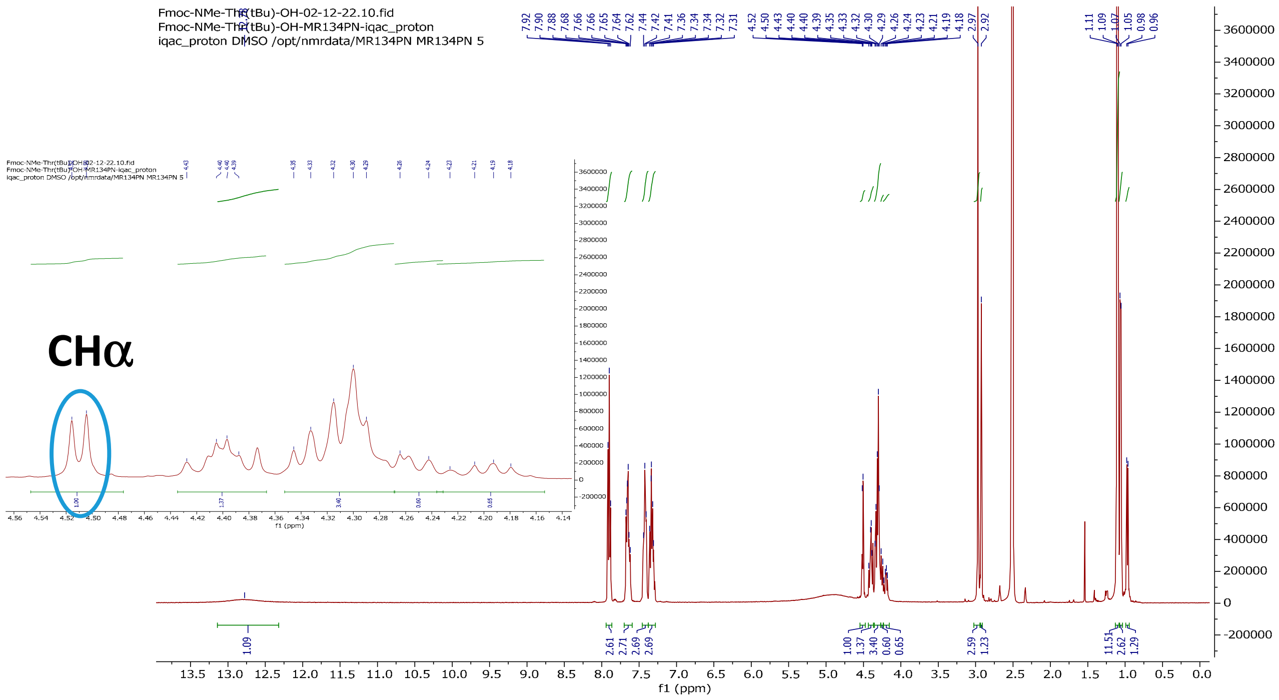

Figure S10: NMR ^{1}H spectrum of Fmoc-NMe-Thr(tBu)-OH enlarged aliphatic zone.

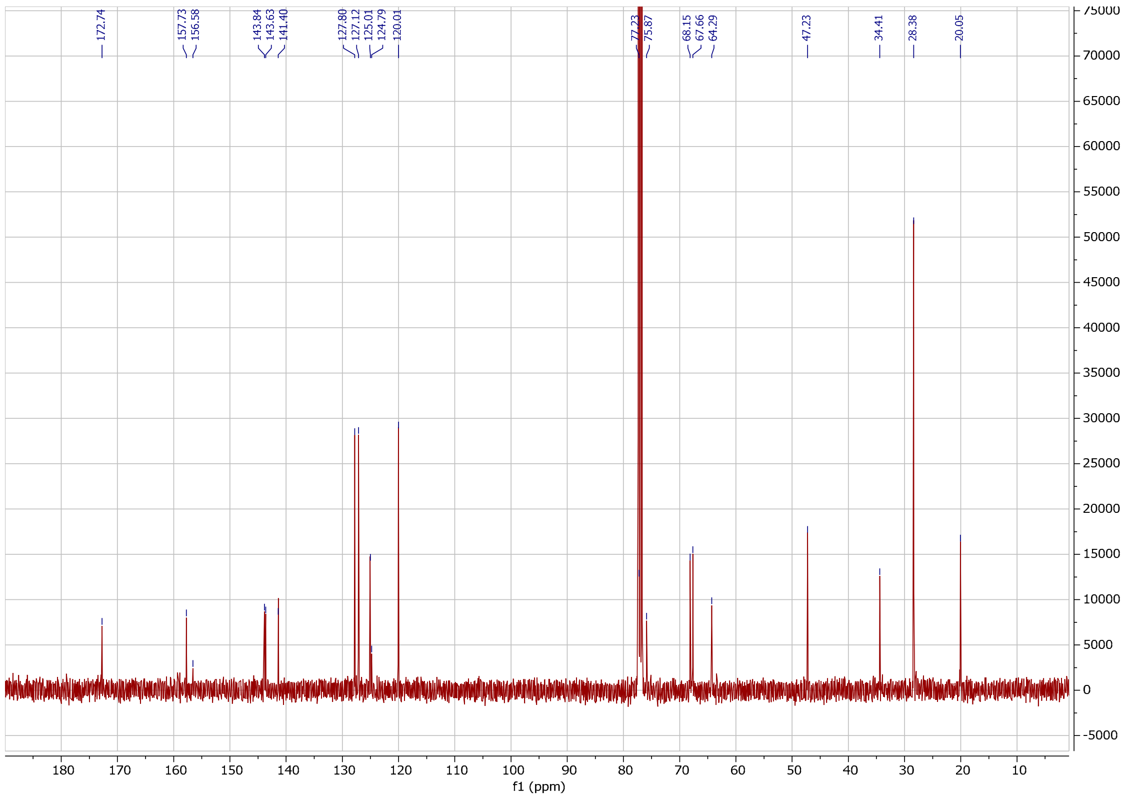

Figure S11: NMR ^{13}C spectrum of Fmoc-N-Me-Thr(tBu)-OH at 400 MHz in $CDCl_3$.

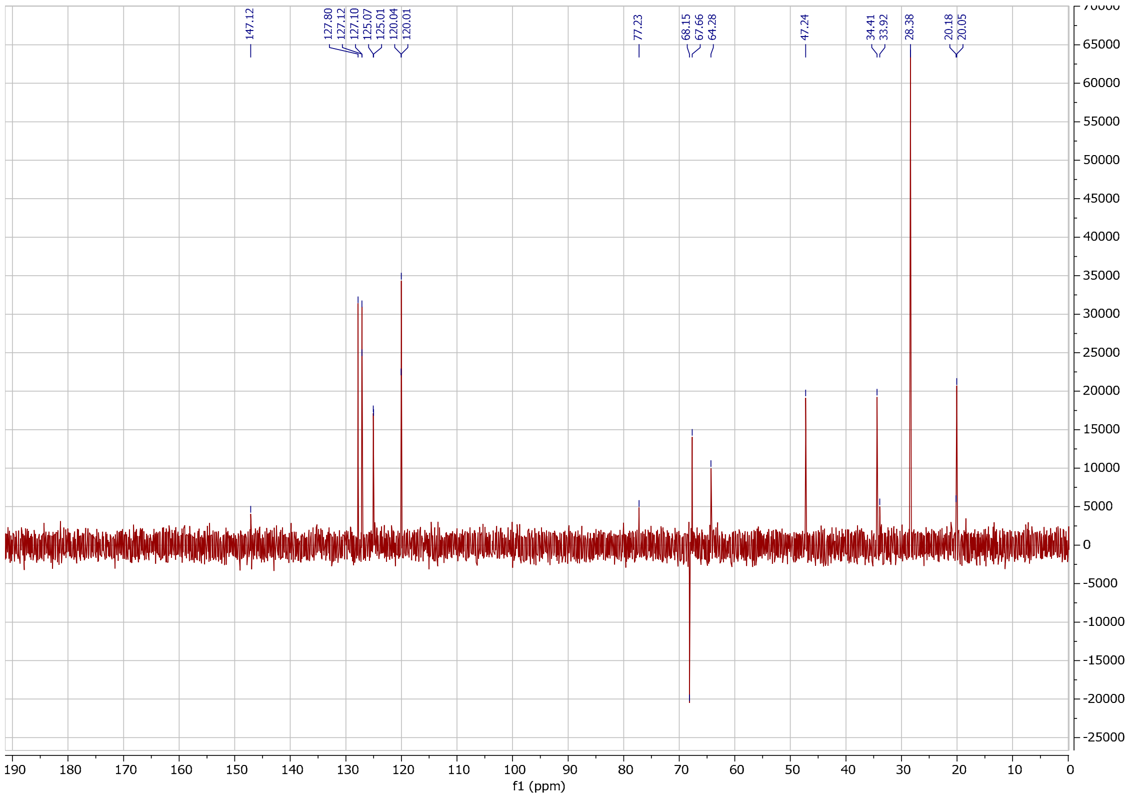

Figure S12: NMR DEP 135 spectrum of Fmoc-N-Me-Thr(tBu)-OH at 400 MHz in $CDCl_3$.

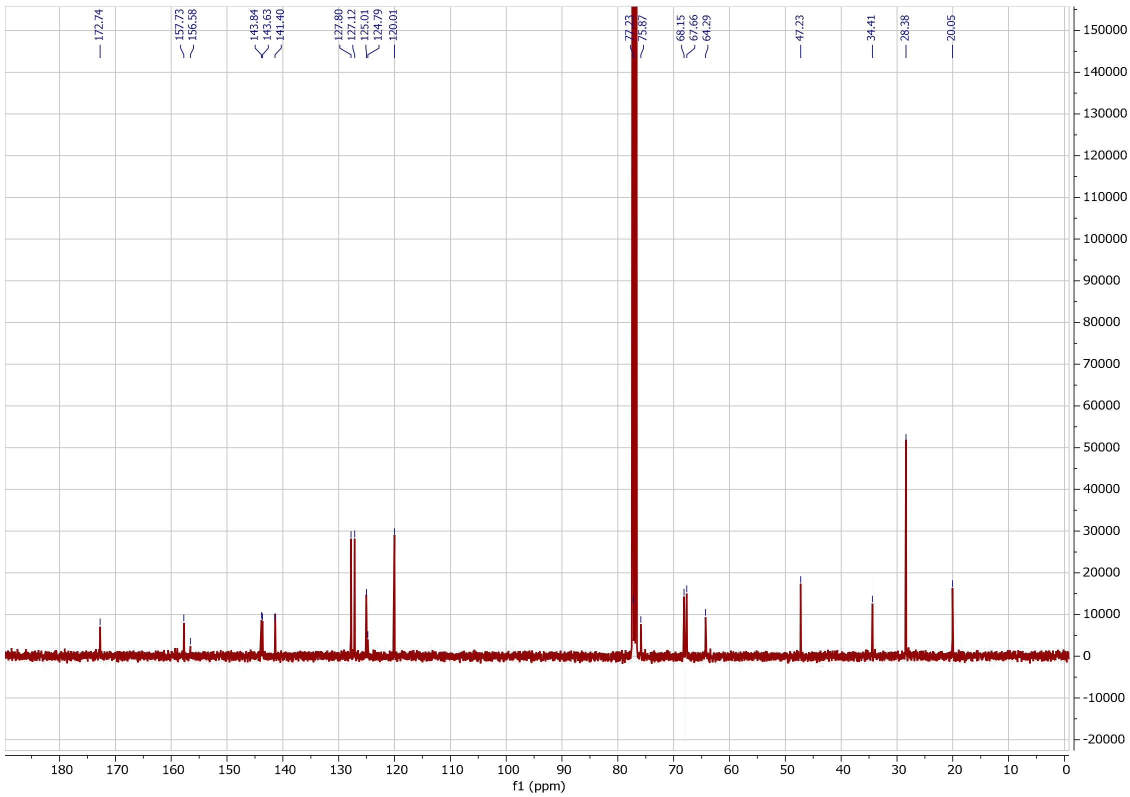

Figure S13: Overlaid ^{13}C and DEP 135 spectra of Fmoc-N-Me-Thr(tBu)-OH at 400 MHz in $CDCl_3$.
